# Implementation of a university faculty mentorship program

**Published:** 2018-11-12

**Authors:** Laura Foxcroft, Douglas Jones, Margaret Steele, Rodrick Lim

**Affiliations:** 1 Department of Medicine, Schulich School of Medicine & Dentistry, Ontario, Canada; 2 Vice Dean of the Department of Basic Medical Sciences, Schulich School of Medicine and Dentistry, Ontario, Canada; 3 Department of Physiology and Pharmacology, Schulich School of Medicine & Dentistry, Ontario, Canada; 4 Dean of the Faculty of Medicine, Memorial University of Newfoundland, Newfoundland and Labrador, Canada; 5 Department of Psychiatry, Memorial University of Newfoundland, Newfoundland and Labrador, Canada; 6 Department of Paediatrics and Medicine, Schulich School of Medicine & Dentistry, Ontario, Canada; 7 Children’s Health Research Institute, Schulich School of Medicine & Dentistry, Ontario, Canada

## Abstract

**Objective:**

To implement a University Faculty mentorship program in the Division of Emergency Medicine.

**Methods:**

A program based on a unique Schulich faculty mentorship policy was implemented with the help of a Provider Value Officer. The process involved creating a training program which defined the roles of the mentors and mentees and established the principles of an effective mentor-mentee relationship. Faculty received training on how to participate effectively in a Schulich faculty mentorship committee. Each committee consisted of a mentee, and two mentors at the associate professor level (one internal and one external). Thirteen distinct external divisions were represented. They were instructed to meet twice per year, as arranged by the mentee. The mentee created mentor minutes using a template, and then submitted the minutes to the members of the mentorship committee and the Chair/Chief of Emergency medicine. The Chair/Chief used the minutes during the annual Continuing Professional Development meeting.

**Results:**

In less than a year, the division has successfully transformed its mentorship program. Using the above-mentioned process, 31 of 34 (91%) eligible assistant professors have functioning mentorship committees. Collaboration and participation between the different faculties has increased. Follow-up meetings with the Chair/Chief and the Provider Value Officer revealed the theme that, universally, participants have perceived Schulich Faculty Mentorship committees as beneficial and are happy with the “fit” of their mentorship committees.

**Conclusion:**

Through careful planning and training, a successful Faculty Mentorship program can be initiated in an academic division in less than a year with the help of a local champion given protected time.

## Introduction

Mentorship is defined as “the process whereby an experienced, highly regarded, empathetic person (the mentor) guides another (usually younger or more junior) individual (the mentee) in the development and re-examination of their own ideas, learning, and personal and professional development.”^1,2^ For clinicians in academic medicine, a robust mentorship program results in greater career and job satisfaction and self-perceived career success.^1,3-5^ Clinicians are also more productive in research, quicker to achieve promotion, and more likely to stay in their institution.^6-11^

Despite these benefits, many institutions have been unable to implement or to sustain mentorship programs due to barriers.^7^ Mentors describe a lack of time or academic credit for participation, a lack of experience with mentorship, and a lack of confidence in their mentoring ability.^7-9^ Mentees’ barriers include lack of time, lack of available well “matched” mentors, and lack of previous mentee experience. Institutionally, barriers include the lack of support for mentoring, lack of formal training, and lack of resources.^1^

The Division of Emergency Medicine, consisting of 75 physicians, including 40 assistant professors requiring promotion to associate professor for lifetime academic rank, implemented the Faculty Mentorship Committee.

### Components of the Mentorship Committee

The Schulich Faculty Mentorship policy^10^ mandated that in order to provide perspectives and increase collaboration, committees consist of three members: the mentee, a mentor from the home division at the associate level, and a member from outside the home department. Department leaders are not ideal mentors due to their conflict of interest, fiduciary roles, and the power differential. Western University is one of the first academic centres in Canada with a policy stating that all faculty members who have not yet achieved career rank are to be offered a mentorship committee.

## Methods

The key pillars adopted to initiate and sustain a successful mentoring program were: to facilitate health for faculty, to develop interfaculty relationships, and to ensure that institutions are positioned to foster success in their faculty.^11^

To promote development of interfaculty relationships, both mentees and mentors attended a two-hour training program, accredited for CME credits. The Chair/Chief mandated training as a prerequisite in the Faculty Mentorship Committee. The Value Provider Officer and a representative from the Dean’s office designed and conducted the training program. They described the Mentorship Program, defined the roles of the mentors and mentees, established the principles of an effective mentor-mentee relationship, and trained faculty to participate effectively. Faculty had the option between multiple dates and times to facilitate attendance at the two-hour training session. After a faculty retreat identified physician wellness and promotion success as a key pillar to division success, the Emergency Division created the Provider Value Officer role. The Provider Value Officer role has protected time. The Provider Value Officer’s first task focuses on faculty mentorship and promotion.

In the summer, the Provider Value Officer created mentorship committees from within the division with each committee consisting of a mentee, a mentor at the associate professor level from within the Division, and an external clinical division at Schulich. The external members represented 13 distinct divisions. Mentees also attended an existing CPD workshop entitled “How to Get Promoted.”

In the fall, the mentees arranged their first mentorship committee meetings. They used a template created to record the minutes (Appendix A). By January, all mentees had submitted minutes from their initial mentorship committee meetings to the Chair/Chief. The minutes guided the Career Development Planning process. As this was an internal quality improvement measure, REB/IRB waived the requirement for consent.

## Results

The Division of Emergency Medicine successfully implemented a University Faculty Mentorship Program in less than a year. Thirty-one of 34 (91%) eligible assistant professors had active mentorship committees. Within six months of this broad initiative, 30 mentorship committees had met and submitted their mentorship meeting minutes to the Chair/Chief. Follow-up meetings with the Chair/Chief and the Provider Value Officer revealed participants universally perceived Schulich Faculty Mentorship Committees as beneficial and were happy with the “fit” of their mentorship committees.

## Future Directions

The Division of Emergency Medicine is developing mentorship committees for all associate professors who would like to have a committee as well as a new pilot workshop called “How to Get Promoted to Full Professor.”

### Conclusion

The implementation of a strong mentorship program in less than a year is possible but requires investment from both faculty and institutions. In the case of the Schulich Mentorship Program, this required a faculty member to champion the cause with identified objectives and protected time. The Faculty-wide policy regarding mentorship provided the incentive and support for the development of this program. The implementation and execution of a strong mentorship program also required training for all faculty, as well as clear guidelines for how meetings should unfold and for how reporting of meetings should occur.
